# Mechanisms of resistance to PARP inhibitors in advanced epithelial ovarian cancer: biological determinants and therapeutic implications

**DOI:** 10.3389/fonc.2026.1892991

**Published:** 2026-09-16

**Authors:** Dalila Incognito, Giuliana Ciappina, Giuseppe Ettore, Carla Ettore, Giosuè Giordano Incognito, Patrizia Carroccio, Emanuela Esposito, Andrea D’Arienzo, Chiara Barraco, Marco Donato, Stefano Cianci, Barbara Busà, Giordana Di Mauro, Massimiliano Berretta

**Affiliations:** 1 Medical Oncology Unit, Department of Human Pathology “G. Barresi”, School of Specialization in Medical Oncology, University of Messina, Messina, Italy; 2 Department of Clinical and Experimental Medicine, University of Messina, Messina, Italy; 3 Department of Medical Sciences, Section of Experimental Medicine, University of Ferrara, Ferrara, Italy; 4 Department of Chemical, Biological, Pharmaceutical and Environmental Sciences, University of Messina, Messina, Italy; 5 Obstetrics and Gynecology Unit, Maternal Child Department, ARNAS Garibaldi, Catania, Italy; 6 Oncology Unit, S. Maria Delle Grazie Hospital, Pozzuoli, Italy; 7 Division of Medical Oncology, AOU “G.Martino” Hospital, University of Messina, Messina, Italy; 8 Obstetrics and Gynecology Unit, Department of Human Pathology of Adult and Childhood “G. Barresi”, University of Messina, Messina, Italy; 9 Pharmacy Unit, University Hospital “G. Martino”, Messina, Italy

**Keywords:** DNA repair, homologous recombination, ovarian carcinoma, poly(ADP-ribose) polymerase inhibitors, drug resistance, recombinational DNA repair

## Abstract

PARP inhibitors have become an integral component of treatment strategies for advanced epithelial ovarian cancer, particularly in *BRCA1/2*-mutated and homologous recombination-deficient (HRD) tumors. Despite clinically meaningful improvements in progression-free survival, a substantial proportion of patients develop primary or acquired resistance, ultimately limiting the long-term efficacy of these agents. Increasing evidence indicates that resistance to PARP inhibition is a biologically heterogeneous process involving restoration of homologous recombination (HR) through *BRCA1/2* reversion mutations, replication fork stabilization, alterations in *PARP1* function, epigenetic reprogramming, drug efflux mechanisms, and adaptive changes in DNA damage response pathways. In parallel, intratumoral heterogeneity and tumor microenvironment remodeling may further contribute to therapeutic failure and clonal selection under treatment pressure. The growing complexity of resistance biology has highlighted the limitations of static genomic biomarkers and has stimulated interest in dynamic molecular profiling approaches, including circulating tumor DNA analysis and functional assays evaluating homologous recombination proficiency (HRP). Several therapeutic strategies are currently being investigated to overcome PARP inhibitor resistance, including combinations with antiangiogenic agents, immune checkpoint inhibitors, *ATR*, *WEE1* and *CHK1* inhibitors, antibody-drug conjugates, and epigenetic therapies. This narrative review summarizes the current understanding of the molecular mechanisms underlying resistance to PARP inhibitors in advanced epithelial ovarian cancer and discusses their potential therapeutic implications, with particular emphasis on biomarker-driven treatment strategies and translational approaches aimed at improving patient selection and long-term disease control.

## 1 Introduction

Ovarian cancer remains one of the leading causes of gynecologic cancer-related mortality worldwide, largely due to the absence of effective screening strategies and the frequent diagnosis at advanced stages of disease. Approximately 70% of ovarian cancers are diagnosed in FIGO stage III–IV, contributing to poor long-term survival despite advances in cytoreductive surgery and platinum-based chemotherapy (1). Epithelial ovarian cancer is characterized by high rates of recurrence and progressive acquisition of therapeutic resistance, representing a major challenge in contemporary gynecologic oncology.

In recent years, increasing attention has been directed toward the molecular landscape of ovarian cancer, particularly alterations involving homologous recombination repair (HRR) pathways. HRR is a high-fidelity DNA repair mechanism responsible for maintaining genomic stability through the accurate repair of double-strand DNA breaks (2). Defects affecting key HRR-related genes, including *BRCA1*, *BRCA2*, *RAD51*, and *PALB2*, impair DNA repair capacity and promote genomic instability, leading to the HRD phenotype (3). Approximately 50% of high-grade serous ovarian cancers (HGSOCs) exhibit HRD-related molecular alterations, making HRD a central biomarker for therapeutic stratification in this disease (4).

The therapeutic implications of HRD have become particularly evident with the clinical development of poly(ADP-ribose) polymerase inhibitors (PARPi). PARPi exploit the concept of synthetic lethality by targeting tumor cells already deficient in HRR mechanisms. In HRR-deficient cells, PARP inhibition results in the accumulation of unrepaired DNA damage, replication fork collapse, and ultimately cell death (2). Several pivotal phase III trials established PARPi maintenance therapy across different clinical settings. SOLO1 evaluated olaparib as first-line maintenance in patients with newly diagnosed advanced ovarian cancer harboring a *BRCA1/2* mutation (5). PRIMA assessed niraparib as first-line maintenance in patients with newly diagnosed advanced ovarian cancer at high risk of recurrence, regardless of biomarker status, although the greatest benefit was observed in the HRD-positive population (6). PAOLA-1 investigated olaparib combined with bevacizumab as first-line maintenance following platinum-based chemotherapy plus bevacizumab, with the most pronounced benefit reported in patients with HRD-positive tumors. In the recurrent setting, ARIEL3 evaluated rucaparib maintenance in patients with platinum-sensitive ovarian cancer who had achieved a response to their most recent platinum-based regimen (7, 8). Collectively, these trials established PARPi as a key component of maintenance treatment in both newly diagnosed and recurrent ovarian cancer. Despite these advances, the clinical implementation of HRD-directed strategies remains complex. Not all HRD-positive tumors derive the same magnitude of benefit from PARPi, while a subset of HR-proficient tumors may still demonstrate partial therapeutic sensitivity. In addition, acquired resistance frequently develops during treatment. Proposed mechanisms include BRCA reversion mutations, restoration of HRP, replication fork stabilization, and activation of compensatory DNA repair pathways (2). These observations have highlighted the limitations of static genomic biomarkers and reinforced the need for more dynamic and biologically informative approaches to HRD assessment.

Current diagnostic strategies integrate *BRCA1/2* mutational analysis with genomic instability metrics such as loss of heterozygosity, telomeric allelic imbalance, and large-scale state transitions (2). However, variability among testing platforms, evolving tumor biology, and the lack of standardized approaches in recurrent disease continue to represent important unresolved issues in clinical practice. In this context, a deeper understanding of HRD biology, mechanisms of PARPi sensitivity and resistance, and currently available diagnostic assays is essential to optimize patient selection and refine therapeutic strategies in epithelial ovarian cancer. This narrative review summarizes the current evidence regarding the molecular basis of HRD, mechanisms of resistance to PARP inhibition, and emerging strategies aimed at improving biomarker-driven treatment approaches in epithelial ovarian cancer.

## 2 Biological basis of PARP inhibitor sensitivity

HRR is a high-fidelity DNA damage repair pathway responsible for the accurate resolution of double-strand DNA breaks, particularly during the S and G2 phases of the cell cycle, when sister chromatids are available as repair templates. Central components of this machinery include *BRCA1* and *BRCA2*, which coordinate DNA end resection, *RAD51* loading, replication fork stabilization, and accurate genomic restoration following genotoxic stress. Loss of HRR function results in genomic instability, chromosomal rearrangements, and progressive accumulation of DNA damage, representing a hallmark of HGSOC (9, 10).

Approximately 40–50% of HGSOCs exhibit HRD, a broader molecular phenotype extending beyond *BRCA1/2* alterations alone. In addition to germline or somatic BRCA mutations, HRD may arise through pathogenic alterations involving *RAD51*C, *RAD51*D, *PALB2*, ATM, *ATR*, CHEK1/2, BRIP1, FANCA, and other HR-related genes, as well as through epigenetic silencing mechanisms such as *BRCA1* promoter methylation. These defects collectively impair HR functionality and force tumor cells to rely on alternative error-prone DNA repair pathways, including non-homologous end joining, thereby promoting genomic instability and driving tumor progression (9, 10).

The therapeutic exploitation of HRD emerged with the development of PARPis, which selectively target tumor cells harboring defective HR through the principle of synthetic lethality. *PARP1* and *PARP2* are critical mediators of single-strand break repair through the base excision repair pathway. Pharmacologic inhibition of PARP activity leads to accumulation of unrepaired single-strand lesions, which are subsequently converted into double-strand breaks during DNA replication. In HR-proficient cells, these lesions can be repaired effectively; conversely, HR-deficient tumor cells accumulate irreparable DNA damage, ultimately resulting in replication fork collapse, mitotic catastrophe, and apoptosis (10).

Beyond catalytic inhibition, PARPis exert cytotoxic effects through PARP trapping, a mechanism whereby PARP enzymes remain physically bound to damaged DNA, generating highly toxic DNA–protein complexes that interfere with replication fork progression and DNA repair machinery recruitment. Among currently available agents, olaparib, niraparib, and rucaparib differ in their trapping potency and pharmacodynamic profiles, although all have demonstrated substantial clinical activity in ovarian cancer maintenance settings (10, 11).

The biological concept of “*BRCA*ness” further expanded the therapeutic applicability of PARPis beyond *BRCA*-mutated tumors. This phenotype describes tumors lacking *BRCA* mutations but exhibiting molecular and functional characteristics similar to BRCA-deficient cancers, including defective HRR, genomic instability, and enhanced sensitivity to platinum compounds and PARP inhibition. Consequently, HRD has become a central predictive biomarker in ovarian cancer, guiding treatment selection and maintenance strategies in both newly diagnosed and recurrent disease settings (9, 11).

Clinically, the introduction of PARPis has profoundly transformed the management of advanced epithelial ovarian cancer. Multiple phase III trials demonstrated significant progression-free survival benefits with maintenance PARPi therapy, particularly among patients with BRCA-mutated or HRD-positive tumors, establishing PARP inhibition as a cornerstone of precision oncology in HGSOC (11).

Given the molecular heterogeneity of HRD, several genomic approaches have been developed to identify tumors more likely to benefit from PARP inhibition. Current HRD assays integrate genomic “scar” signatures, including loss of heterozygosity, telomeric allelic imbalance, and large-scale state transitions, which collectively reflect historical defects in HRR. Additional strategies include mutational signatures such as COSMIC Signature 3, *RAD51* functional assays, and emerging multi-omics approaches integrating epigenetic and transcriptomic data (9).

Despite these advances, HRD testing remains imperfect and biologically dynamic. Current genomic scar assays may fail to identify tumors with restored HR function or emerging resistance mechanisms, highlighting the limitations of static biomarkers in a continuously evolving genomic landscape. These challenges have stimulated growing interest in functional HRD assays, circulating tumor DNA monitoring, and real-time molecular profiling strategies capable of capturing both intrinsic and acquired resistance during PARPi exposure (10, 12).

## 3 Mechanisms of resistance to PARP inhibitors

PARP inhibitor sensitivity is not determined only by catalytic inhibition of *PARP1*/2, but also by the ability of these agents to trap *PARP1* on damaged chromatin, thereby converting DNA repair intermediates into cytotoxic DNA-protein complexes (13). Following PARPi exposure, trapped *PARP1* obstructs DNA repair, interferes with replication fork progression, and amplifies replication-associated DNA damage, particularly in tumors with defective HRR (14). This mechanism is clinically relevant because reduced *PARP1* trapping, either through *PARP1* loss, *PARP1* alterations, or modulation of *PARP1* chromatin binding, has been recognized as a potential route to PARPi resistance in ovarian cancer (14, 15). Recent evidence suggests that ovarian cancer cells may attenuate *PARP1* trapping through non-canonical chromatin regulatory mechanisms, as shown by KAT6A liquid-liquid phase separation, which sequesters *PARP1* away from DNA break sites and reduces the cytotoxic effect of PARPi (13). Similarly, *HMGB3* has been shown to promote PARPi resistance by interacting with *PARP1*, whereas *HMGB3* depletion increases *PARP1* trapping at DNA lesions, suppresses PARylation activity, enhances DNA damage signaling, and restores sensitivity to PARPi in ovarian cancer models (16). Replication stress represents another central determinant of PARPi activity, because stalled or collapsed replication forks can convert unresolved single-strand lesions into double-strand breaks and increase dependence on checkpoint-mediated DNA damage response pathways (17, 18). HGSOC is intrinsically characterized by high levels of endogenous replicative stress because of universal *TP53* dysfunction, genomic instability, copy-number alterations, and frequent disruption of cell-cycle checkpoints, which together increase reliance on the *ATR*-*CHK1*-*WEE1* axis for survival under genotoxic pressure. The *ATR*-*CHK1*-*WEE1* axis coordinates replication fork stabilization, S-G2 checkpoint activation, and DNA repair, thereby enabling tumor cells to tolerate PARPi-induced genomic stress (17). In PARPi-resistant BRCA-mutated HGSOC models, increased *ATR*/*CHK1* signaling has been associated with restoration of replication stress tolerance and therapeutic escape, supporting the rationale for targeting this pathway in resistant disease (19). R-loop accumulation has also emerged as a relevant source of replication stress in ovarian cancer, and the interaction between AKT1 and DHX9 appears to facilitate R-loop resolution, thereby limiting pathological replication stress in PARPi-resistant BRCA-mutated HGSOC (20). Dual *ATR* and AKT inhibition was shown to increase R-loop-mediated replication stress, DNA damage, and apoptosis in both PARPi-sensitive and PARPi-resistant BRCA-mutated HGSOC models, suggesting that forced replication stress may represent a rational strategy to overcome resistance (11, 19). Additional evidence supporting replication stress rewiring derives from studies showing that *CHK1* inhibition induces cell-cycle dysregulation, HR impairment, and excessive DNA damage, whereas combined *CHK1* and PARG inhibition enhances PARylation, NAD+ depletion, and mitotic catastrophe in ovarian cancer models (20). Low *PPP2R2A* expression has also been linked to increased c-*MYC*-driven replication initiation, heightened replication stress, and greater dependence on *CHK1* signaling, identifying *PPP2R2A* deficiency as a potential biomarker of *CHK1* inhibitor sensitivity in HGSOC, including PARPi-resistant settings (21). In parallel, epigenetic rewiring may reinforce PARPi resistance by modifying transcriptional programs involved in DNA repair, stemness, and drug efflux, as illustrated by the *PARP1*-*DOT1L* axis, which promotes *DOT1L* transcription and regulates PLCG2 and *ABCB1* through H3K79me2-dependent mechanisms (22). Together, these findings indicate that PARPi resistance cannot be reduced to restoration of HR alone, but should be interpreted as an adaptive state involving impaired PARP trapping, replication fork protection, checkpoint kinase dependency, R-loop resolution, metabolic stress adaptation, and epigenetic transcriptional remodeling (14). This biological framework provides the rationale for therapeutic strategies aimed at overwhelming resistant tumor cells with unrepaired replication stress, particularly through combinatorial approaches involving *ATR*, *CHK1*, *WEE1*, PARG, AKT, or *DOT1L*-directed combinations (19, 20).

### 3.1 Restoration of homologous recombination and replication fork protection

HRD represents the biological basis underlying the synthetic lethality induced by PARPi, and restoration of HR has emerged as one of the principal mechanisms responsible for acquired resistance in ovarian cancer (14). In HR-proficient cells, DNA double-strand breaks are repaired through a highly coordinated pathway involving DNA end resection, recruitment of *BRCA1/2* and *PALB2*, and *RAD51*-mediated strand invasion, ultimately restoring genomic integrity with high fidelity (14).

PARPi resistance frequently develops through restoration of HR competency, thereby enabling tumor cells to repair replication-associated DNA lesions despite ongoing PARP inhibition (10).

Secondary reversion mutations affecting *BRCA1*, *BRCA2*, and *RAD51* family genes can restore the open reading frame and partially recover HR function, thereby reducing sensitivity to PARPi and platinum-based chemotherapy (23, 24). This biological overlap explains the frequent cross-resistance observed between PARPi and platinum agents, as both therapeutic approaches exploit HRD to induce lethal DNA damage and depend on defective DNA repair for their antitumor activity. Restoration of HR through *BRCA1/2* reversion mutations, *RAD51* reactivation, and replication fork protection represents one of the principal mechanisms driving resistance to both treatment modalities (25). By contrast, alterations affecting *PARP1* function, including mutations that impair PARP trapping or PARylation without restoring HR, are considered predominantly PARPi-specific mechanisms and may not necessarily compromise platinum sensitivity. In addition, replication fork stabilization can contribute to cross-resistance by protecting stalled replication forks from nucleolytic degradation, thereby limiting the cytotoxic effects of both PARPi and platinum compounds even in tumors that remain partially HR-deficient. Recognizing the biological differences between shared and PARPi-specific resistance mechanisms may improve therapeutic sequencing and support the rational development of biomarker-guided combination strategies aimed at delaying or overcoming treatment resistance (17).

The restoration of HR is closely linked to the recovery of *RAD51* loading onto damaged DNA, a critical step enabling homologous strand invasion and D-loop formation during DNA repair (14).

*RAD51* has therefore emerged as a dynamic functional biomarker of HR proficiency and PARPi resistance beyond BRCA mutational status, reflecting real-time restoration of DNA repair capacity under therapeutic pressure (14). Although *RAD51* functional assays have shown considerable promise for identifying HRP and predicting PARPi sensitivity, their use remains largely confined to the research setting. Further prospective validation and assay standardization are required before routine implementation in clinical practice (26).

Several adaptive molecular programs can indirectly restore HR signaling in initially HR-deficient tumors. Epigenetic regulators, chromatin remodeling pathways, and transcriptional networks have all been implicated in reactivating *BRCA1/2* and *RAD51* expression (27).

LSD1 has recently been identified as an important regulator of HR proficiency in ovarian cancer, and its inhibition was shown to impair *BRCA1/2* and *RAD51* transcription, thereby inducing an HR-deficient phenotype and sensitizing HR-proficient ovarian cancer cells to PARPi (27).

Similarly, the long non-coding RNA NEAT1 contributes to PARPi resistance by sustaining *RAD51* expression and HR repair activity in serous ovarian cancer cells (28).

NEAT1 silencing reduced *RAD51* and FOXM1 expression, impaired HR efficiency, increased γ-H2AX accumulation, and enhanced olaparib-induced DNA damage, ultimately restoring PARPi sensitivity (28).

Emerging evidence also indicates that post-translational modifications regulating HR proteins may contribute to therapeutic resistance. Histone H3K9 lactylation and *RAD51* lactylation have been shown to enhance HR repair activity and promote platinum resistance in ovarian cancer models (28, 29).

Mechanistically, H3K9 lactylation directly increased *BRCA2* and *RAD51* transcription, whereas *RAD51*K73 lactylation enhanced HR repair efficiency, supporting tumor survival under genotoxic stress (28). Beyond HR restoration, stabilization of stalled replication forks has emerged as an additional and partially independent mechanism of PARPi resistance.

Replication fork protection allows tumor cells to prevent excessive nucleolytic degradation during replication stress, thereby limiting genomic instability and promoting survival despite persistent HR defects (23). *PARP1* and *BRCA2* cooperate in the stabilization of *RAD51* at stalled replication forks and protect nascent DNA from MRE11-mediated degradation (23, 29).

Loss of factors involved in fork degradation, including PTIP, CHD4, EZH2, and MRE11 regulatory pathways, can restore fork protection and confer resistance to both PARPi and platinum agents in BRCA-deficient tumors. Importantly, replication fork stabilization may occur even in the absence of full HR restoration, suggesting that fork protection and HR recovery represent biologically distinct but functionally complementary resistance mechanisms (23). These findings highlight the remarkable plasticity of ovarian cancer cells under selective therapeutic pressure and underscore the need for combinatorial strategies capable of simultaneously targeting HR restoration, replication fork protection, and adaptive DNA damage response signaling.

### 3.2 *PARP1* alterations, drug efflux, and epigenetic reprogramming

Beyond restoration of HR and replication fork stabilization, ovarian cancer cells may develop PARP inhibitor resistance through a broad spectrum of adaptive mechanisms involving *PARP1* functional alterations, drug efflux activation, chromatin remodeling, and transcriptional reprogramming. These processes collectively contribute to the emergence of dynamic resistant phenotypes capable of surviving sustained therapeutic pressure despite persistent genomic instability (14).

*PARP1* plays a central role in the DNA damage response through recognition of DNA strand breaks, recruitment of repair complexes, regulation of chromatin accessibility, and stabilization of stalled replication forks (30). The cytotoxic activity of PARP inhibitors depends not only on catalytic inhibition of PARylation but also on the ability to trap *PARP1* on damaged chromatin, thereby generating toxic PARP–DNA complexes that interfere with replication fork progression and DNA repair signaling (30). Consequently, alterations reducing *PARP1* trapping efficiency, impairing *PARP1* chromatin binding, or decreasing cellular dependence on PARP-mediated repair pathways may significantly attenuate therapeutic efficacy (14).

Accumulating evidence indicates that resistant ovarian cancer cells undergo extensive transcriptional and epigenetic rewiring under PARPi-induced selective pressure. Chromatin remodeling programs may reactivate HR-associated genes, modulate replication stress responses, and alter accessibility of DNA repair machinery, thereby promoting adaptive resistance states even in tumors initially classified as HR-deficient (30). These observations support the concept that HRD should not be interpreted as a static molecular condition, but rather as a dynamic and evolutionarily plastic phenotype that may fluctuate during treatment exposure.

Epigenetic plasticity also appears to contribute to broader transcriptional adaptation programs associated with tumor survival, stemness, and metabolic rewiring. Recent studies demonstrated that SUMOylation-dependent signaling pathways sustain survival of *MYC*-driven ovarian cancer cells and facilitate adaptation to replication-associated stress (31). In resistant models, transcriptional profiling revealed enrichment of stress-response pathways, altered chromatin regulatory networks, and increased expression of genes involved in multidrug resistance and cellular detoxification programs (31).

Drug efflux activation represents another major mechanism limiting PARPi efficacy. ATP-binding cassette transporters, particularly *ABCB1* and *ABCG2*, have been consistently implicated in reduced intracellular drug accumulation and multidrug resistance phenotypes in ovarian cancer (32). Experimental models resistant to niraparib demonstrated marked induction of *ABCG2* expression, whereas *ABCB1* upregulation was more frequently associated with prior taxane exposure and cross-resistance patterns (23, 32). Functional silencing of *ABCG2* restored PARPi sensitivity, while pharmacologic inhibition of efflux transporters partially reversed resistant phenotypes, supporting the direct contribution of transporter-mediated drug extrusion to therapeutic escape (32, 33).

The biological complexity of adaptive resistance is further reinforced by the remarkable molecular heterogeneity of ovarian cancer. Multi-omics analyses have demonstrated that ovarian tumors may dynamically activate distinct signaling pathways involving epithelial-mesenchymal transition, inflammatory signaling, metabolic adaptation, and stress-response networks during disease progression and treatment exposure (34). These findings suggest that PARPi resistance should be viewed as an evolving ecosystem-level process involving coordinated interactions between genomic instability, epigenetic remodeling, metabolic plasticity, and clonal selection rather than as a consequence of isolated molecular events alone (23).

Importantly, adaptive resistance mechanisms may emerge in parallel within distinct tumor subclones, contributing to substantial intratumoral heterogeneity and temporal variability in treatment response. Such evolutionary dynamics likely explain why static genomic assays may inadequately capture the biologic complexity of resistant disease. In this context, increasing attention has focused on longitudinal molecular profiling approaches, including circulating tumor DNA analysis and functional assays capable of dynamically assessing HRP over time (2).

Collectively, these observations indicate that PARP inhibitor resistance extends beyond restoration of HR alone and instead reflects a highly adaptive biological state driven by *PARP1* rewiring, epigenetic plasticity, transcriptional adaptation, drug efflux activation, and clonal evolutionary pressure. This evolving understanding provides the rationale for therapeutic strategies combining PARP inhibitors with agents targeting chromatin remodeling, *ATR*/*CHK1* signaling, epigenetic regulators, or multidrug resistance pathways to suppress adaptive tumor plasticity and restore therapeutic sensitivity.

### 3.3 Tumor microenvironment, intratumoral heterogeneity, and clonal evolution

PARPi resistance should not be interpreted exclusively as a tumor cell-intrinsic process, because ovarian cancer evolves within a spatially organized ecosystem composed of malignant subclones, stromal cells, immune populations, and treatment-shaped cellular niches (35). Intratumoral heterogeneity may dynamically change during treatment, with chemotherapy and PARPi exposure selecting resistant clones while suppressing more sensitive cellular populations. This temporal plasticity is supported by observations that HRD and LOH patterns may fluctuate between primary, residual, and recurrent tumors, indicating that HRD status can reflect treatment-driven clonal selection rather than a fixed molecular state (35).

From an evolutionary perspective, continuous drug exposure may create selective pressure favoring pre-existing or newly emerging resistant subpopulations, including clones with restored HR, enhanced replication fork protection, altered drug transport, or microenvironment-dependent survival advantages (36). Mathematical and experimental modeling of olaparib scheduling suggests that treatment dynamics are strongly influenced by timing, dose modulation, and the persistence of sensitive populations, supporting the concept that resistance may arise from ecological competition between drug-sensitive and drug-tolerant clones (34, 36).

Cancer stemness represents another layer of intratumoral heterogeneity relevant to PARPi resistance. PARPi-resistant ovarian cancer cells have been shown to display increased stemness features, including higher expression of SOX2, KLF4, Nanog, and OCT4, enhanced sphere-forming capacity, and increased ALDH activity (37). *KLF5* appears to promote this resistant phenotype by regulating Vimentin and sustaining stemness-associated programs, whereas *KLF5* inhibition reduced stemness features and restored sensitivity to PARPi in preclinical ovarian cancer models (37, 38).

Cellular senescence and mitochondrial quality control may also contribute to adaptive resistance. Inhibition of lncRNA PART1 was associated with resistance to both cisplatin and PARPi, promotion of cellular senescence, degradation of PHB2, impaired mitophagy, and reduced treatment sensitivity. These findings suggest that senescent-like resistant states may support tumor persistence under genotoxic pressure and may represent a non-canonical mechanism of PARPi escape (39).

The tumor microenvironment has emerged as a critical determinant of PARPi response. Preclinical and translational evidence indicates that PARPi exposure may remodel immune and stromal compartments, particularly through macrophage polarization, interferon signaling, *STAT3* activation, and suppression of cytotoxic antitumor immunity (40). In *BRCA1*-deficient ovarian cancer models, acquired PARPi resistance was associated with enrichment of protumor macrophages driven by PARPi-induced *STAT3* signaling in tumor cells, while *STING* agonism reprogrammed myeloid cells and restored PARPi sensitivity (40).

Recent spatial profiling studies have further refined this concept by showing that PARPi-resistant lesions may contain IFN-positive tumor cell niches characterized by intensified immunosuppressive interactions (41). In treatment-naive HGSOC exposed to neoadjuvant niraparib, persistent TAM enrichment was associated with nonresponse, while high baseline SPP1-positive cells predicted lower response rates and shorter progression-free survival (41). These data support an immune-dependent resistance model in which tumor-derived IFN sustains SPP1-positive macrophage niches enriched in Tregs and myofibroblastic cancer-associated fibroblasts, ultimately impairing T-cell effector function and promoting therapeutic escape (41).

Conversely, immune effector populations may also represent exploitable vulnerabilities within the ovarian cancer microenvironment. Adaptive NK cells have been shown to exhibit tumor-specific immune memory, preferential infiltration into the ovarian tumor microenvironment, and enhanced cytotoxicity against autologous tumor cells compared with conventional NK cells (42). Although these findings are not specific to PARPi resistance, they further support the biological relevance of immune remodeling in HGSOC and may influence future strategies combining DNA damage-directed therapies with immune-based approaches (42, 43).

Collectively, these findings indicate that PARPi resistance reflects a multilevel adaptive process involving clonal evolution, stemness acquisition, senescence-associated persistence, immune suppression, macrophage reprogramming, and spatial remodeling of tumor-stromal interactions (38). This framework reinforces the limitations of static genomic biomarkers and supports longitudinal profiling approaches capable of integrating tumor-intrinsic alterations with microenvironmental and immune determinants of resistance (44). Therefore, therapeutic strategies aimed at overcoming PARPi resistance will likely require not only targeting HR restoration or replication fork protection, but also disrupting the ecological and immunosuppressive niches that sustain resistant ovarian cancer clones (45).

## 4 Therapeutic strategies to overcome PARPi resistance

Multiple therapeutic strategies are currently under investigation to overcome PARPi resistance by targeting the molecular vulnerabilities emerging during adaptive resistance evolution. Rather than relying exclusively on sequential PARPi administration, current approaches aim to interfere with compensatory DNA damage response pathways, replication fork stabilization, tumor microenvironment remodeling, and alternative survival signaling networks (46).

One of the most extensively explored approaches involves the inhibition of cell cycle checkpoint kinases, particularly *ATR*, *CHK1*, and *WEE1*. These proteins are essential for maintaining G2/M checkpoint integrity and promoting DNA repair under replication stress conditions. In PARPi-resistant tumors, especially those exhibiting partial HR restoration or replication fork protection, checkpoint inhibition may restore sensitivity by forcing cells to progress through the cell cycle despite unresolved DNA damage (23).

*ATR* inhibition has shown particular promise because *ATR* signaling contributes not only to HRR but also to replication fork stabilization. Preclinical models demonstrated that *ATR* blockade increases replication fork collapse, chromosomal instability, and DNA double-strand break accumulation in PARPi-resistant ovarian cancer cells, including BRCA-reverted models (47). In contrast, *CHK1* inhibition alone may be insufficient to fully suppress fork protection mechanisms, suggesting that *ATR* inhibition could provide broader synthetic lethality in resistant tumors (23).

*WEE1* inhibition represents another attractive strategy, particularly in *TP53*-mutated HGSOC, where tumor cells are highly dependent on the G2/M checkpoint. *WEE1* blockade promotes premature mitotic entry in the presence of unrepaired DNA lesions, thereby enhancing replication catastrophe and potentiating PARPi-induced cytotoxicity (30).

Immune-based therapeutic combinations have also emerged as a potential strategy to overcome PARPi resistance. PARP inhibition can increase genomic instability, neoantigen generation, and cytosolic DNA accumulation, thereby activating the cGAS-*STING* pathway and enhancing interferon signaling within the tumor microenvironment. These observations provide the biological rationale for combining PARPi with immune checkpoint inhibitors (46). Early clinical studies combining PARPi with PD-1 or PD-L1 inhibitors have demonstrated encouraging activity, particularly in platinum-sensitive and HR-deficient ovarian cancer subgroups, although predictive biomarkers of response remain poorly defined (46).

Antiangiogenic agents may also restore PARPi sensitivity by inducing hypoxia-mediated HR suppression. Hypoxic conditions reduce *BRCA1* and *RAD51* expression, potentially recreating an HR-deficient phenotype even in tumors that have partially restored HR activity. This biological interaction provides the rationale for combining PARPi with antiangiogenic drugs such as cediranib or bevacizumab (33, 48).

The phase II EVOLVE study evaluated cediranib plus olaparib in patients with HGSOC following progression on previous PARPi therapy. Its translational analysis showed that longitudinal circulating tumor DNA profiling could identify emerging resistance mechanisms, including BRCA1/2 reversion mutations, and reveal molecular alterations not detected in baseline tumor tissue. These findings support the potential use of liquid biopsy as a minimally invasive dynamic tool for monitoring resistance evolution and informing subsequent treatment selection (49).

Increasing attention has recently focused on targeting epigenetic and transcriptional regulators involved in PARPi resistance. Aberrant activation of transcriptional programs sustaining HR and DNA repair has been implicated in adaptive resistance. *SOX9* overexpression, for example, has been associated with olaparib resistance through transcriptional activation of multiple DNA damage repair genes, including SMARCA4, UIMC1, and SLX4 (50). Mechanistically, USP28 stabilizes *SOX9* by preventing its ubiquitin-mediated degradation, thereby promoting DNA repair proficiency and resistance to PARPi. Importantly, pharmacologic inhibition of USP28 restored olaparib sensitivity in preclinical ovarian cancer models, supporting the therapeutic potential of targeting ubiquitin-proteasome regulatory networks (46, 50). Additional strategies aim to exploit acquired dependencies generated during resistance evolution. POLQ inhibition has emerged as a promising synthetic lethal approach in HRD tumors, particularly in cases where residual genomic instability persists despite partial HR restoration. Similarly, proteolysis-targeting chimeras (PROTACs) designed to degrade *PARP1* or DDR-related proteins may overcome resistance mechanisms associated with *PARP1* mutations or restored PARylation activity (46). PARPi rechallenge has also been investigated as a therapeutic strategy after previous exposure to this drug class. In the phase IIIb OReO/ENGOT-ov38 trial, maintenance olaparib rechallenge resulted in a statistically significant, although modest, improvement in progression-free survival compared with placebo in patients with platinum-sensitive relapsed ovarian cancer previously treated with a PARPi. The benefit was observed in both the BRCA1/2-mutated and non-BRCA1/2-mutated cohorts. These findings indicate that previous PARPi exposure does not necessarily result in complete class resistance and highlight the need for biomarkers capable of identifying patients who may retain sensitivity to *PARP* inhibition (51). Collectively, these findings suggest that PARPi resistance should no longer be viewed as a single biological event, but rather as a dynamic and heterogeneous adaptive state characterized by evolving genomic, epigenetic, and microenvironmental alterations. Future therapeutic strategies will likely require biomarker-driven combination approaches capable of simultaneously targeting HR restoration, replication fork protection, and tumor microenvironment-mediated immune escape while limiting the emergence of resistant tumor subclones.

Several combination strategies targeting these resistance mechanisms are currently being evaluated in clinical trials, with the aim of translating preclinical findings into effective therapeutic approaches for patients with advanced ovarian cancer. A summary of selected clinical trials investigating these strategies is provided in **Table 1**.

**Table 1:** Representative clinical trials investigationg therapeutic strategies targeting mechanisms of PARPi resistance in ovarian cancer.

Representative trial	Phase	Investigational regimen	Disease setting	Therapeutic strategy	Targeted mechanism	ClinicalTrials.gov identifier
CAPRI (52)	II	Ceralasertib + olaparib	PARPi-resistant recurrent ovarian cancer	*ATR* inhibition	Replication stress response	NCT03462342
ATARI (53)	II	Ceralasertib ± olaparib (± durvalumab)	Relapsed gynecological cancers*	*ATR* inhibition	Replication stress response	NCT04065269
EFFORT (54)	II	Adavosertib ± olaparib	Recurrent ovarian cancer	*WEE1* inhibition	G2/M checkpoint abrogation	NCT03579316
MEDIOLA (55)	II	Olaparib + durvalumab	Platinum-sensitive BRCA-mutated recurrent ovarian cancer	Immune checkpoint inhibition	cGAS-*STING* activation and immune modulation	NCT02734004
DUO-O (56)	III	Durvalumab + bevacizumab + olaparib	Newly diagnosed advanced ovarian cancer	Immune checkpoint inhibition	Immune modulation and antiangiogenesis	NCT03737643
KEYLYNK-001 (57)	III	Pembrolizumab + chemotherapy ± olaparib	Newly diagnosed advanced ovarian cancer	Immune checkpoint inhibition	Immune modulation	NCT03740165
CONCERTO (58)	II	Cediranib + olaparib	Platinum-resistant ovarian cancer	Antiangiogenic therapy	Hypoxia-induced HR suppression	NCT02889900
OReO (51)	IIIb	Olaparib rechallenge vs placebo	Platinum-sensitive relapsed ovarian cancer previously treated with a PARPi	PARPi rechallenge	Residual PARPi sensitivity following prior exposure	NCT03106987
EVOLVE (49)	II	Cediranib + olaparib	HGSOC after progression on previous PARPi therapy	Antiangiogenic therapy	Post-PARPi resistance and longitudinal ctDNA monitoring	NCT02681237

A schematic overview of emerging therapeutic strategies aimed at restoring PARPi sensitivity in epithelial ovarian cancer is presented in **Figure 1**.

**Figure 1:**
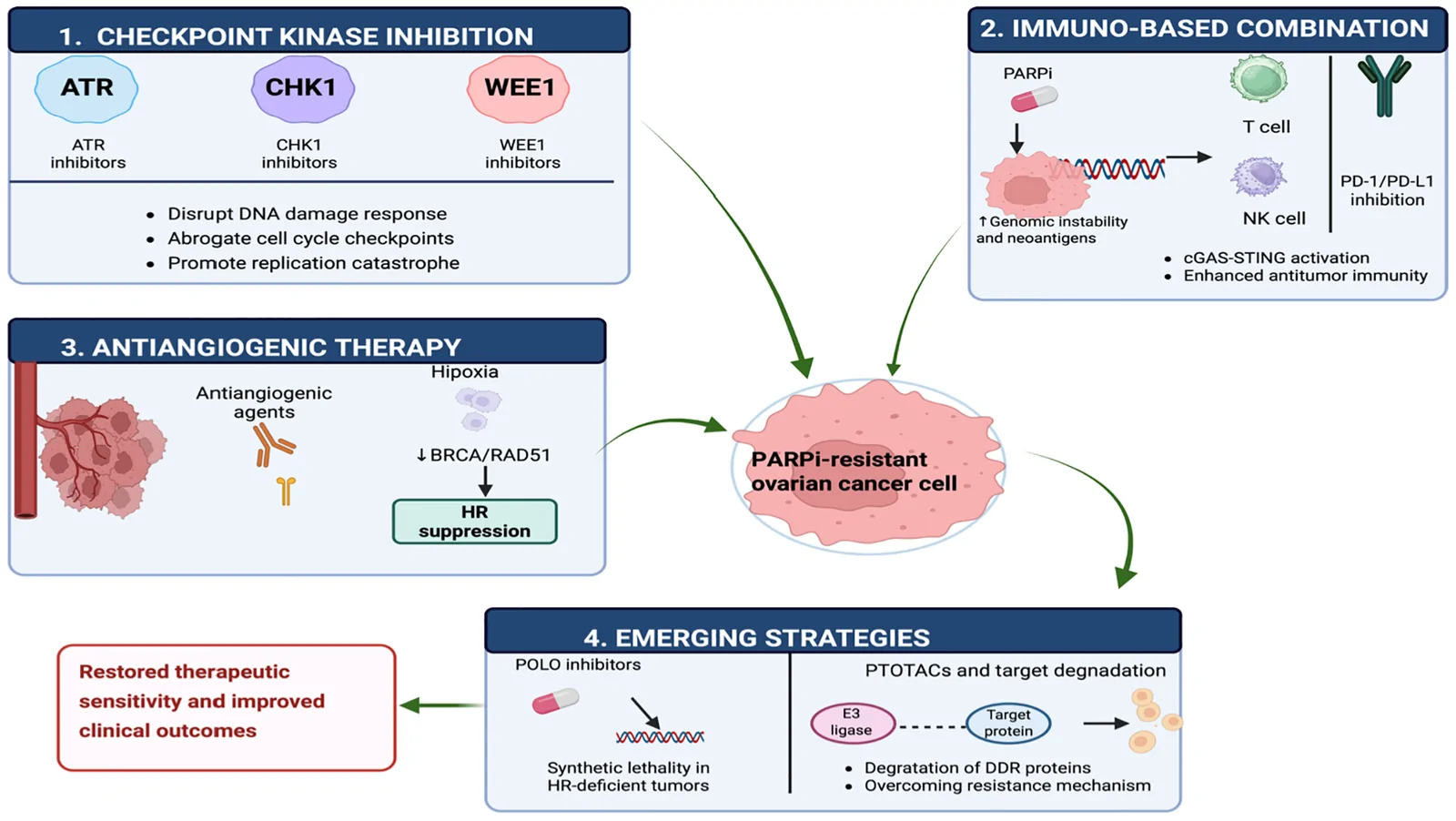
Schematic overview of the main approaches currently under investigation to restore PARPi sensitivity in resistant ovarian cancer. Strategies include *ATR*, *CHK1*, and *WEE1* inhibition to increase replication stress, immune checkpoint blockade and *STING* activation to modulate the tumor microenvironment, antiangiogenic therapies to induce HR suppression, and epigenetic or transcriptional targeting approaches. Additional emerging strategies include POLQ inhibition and PROTAC-based therapies directed against DNA damage response pathways.

## 5 Future perspectives

Future research on PARP inhibitor resistance will likely focus on the integration of dynamic biomarkers, spatial biology, and micro. environmental profiling to better characterize resistance evolution during treatment. Current HRD assays provide only a static representation of HRD and may fail to capture adaptive restoration of DNA repair pathways under therapeutic pressure. In this context, functional HR assays, circulating tumor DNA analysis, and single-cell or spatial transcriptomic approaches may improve real-time assessment of therapeutic sensitivity and clonal evolution (41). Recent clinical studies provide an initial framework for translating these approaches into practice. In EVOLVE, longitudinal circulating tumor DNA profiling at the time of PARPi progression identified emerging resistance alterations, including *BRCA1/2* reversion mutations, supporting liquid biopsy as a dynamic tool for monitoring clonal evolution (49). CAPRI and EFFORT further suggest that acquired dependence on *ATR* and *WEE1* mediated checkpoint signaling may be therapeutically exploited after PARPi resistance, although prospective biomarker selection remains necessary to identify the patients most likely to benefit (43, 54). In OReO, olaparib rechallenge produced a modest progression-free survival benefit in selected patients with platinum-sensitive relapse previously exposed to a PARPi, indicating that resistance may not be absolute and reinforcing the need for biomarkers of residual PARPi sensitivity (51). Beyond PARPi-directed combinations and rechallenge, the management of recurrent ovarian cancer is increasingly moving toward biomarker-selected strategies, including WEE1 inhibition and antibody-drug conjugates targeting FRα or HER2, which may expand treatment options for patients previously exposed to PARPi (59). At the same time, increasing attention is being directed toward combination strategies targeting replication stress pathways, HR restoration, and tumor microenvironment-mediated immune suppression. In particular, the identification of IFN-responsive and osteopontin-enriched macrophage niches in resistant lesions suggests that immune and stromal remodeling may represent clinically relevant therapeutic targets (42). Future biomarker-driven clinical trials integrating molecular and microenvironmental determinants of resistance may therefore improve therapeutic stratification and delay adaptive therapeutic escape in advanced ovarian cancer.

## 6 Conclusion

PARP inhibitors have significantly improved the management of advanced epithelial ovarian cancer, particularly in BRCA-mutated and HRD-positive disease. Nevertheless, the emergence of primary and acquired resistance remains a major clinical challenge limiting the durability of therapeutic benefit. Current evidence indicates that PARPi resistance is a biologically heterogeneous and dynamic process involving restoration of HR, replication fork stabilization, DNA damage response rewiring, epigenetic adaptation, drug efflux activation, and tumor microenvironment remodeling. Increasing recognition of intratumoral heterogeneity and clonal evolution has further highlighted the limitations of static HRD assays and reinforced the need for dynamic biomarkers capable of monitoring tumor evolution during treatment. Functional HR assays, circulating tumor DNA analysis, and multi-omics approaches may improve patient stratification and facilitate earlier identification of resistant disease states.

At the same time, growing understanding of resistance biology has stimulated the development of novel therapeutic strategies aimed at targeting adaptive vulnerabilities in resistant tumors. Combination approaches integrating PARPi with *ATR*, *CHK1*, *WEE1*, immune checkpoint, antiangiogenic, or epigenetic therapies represent promising strategies to restore therapeutic sensitivity and delay adaptive escape.

Overall, overcoming PARPi resistance will likely require biologically integrated and biomarker-driven therapeutic approaches capable of simultaneously targeting DNA repair restoration, replication stress adaptation, and microenvironment-mediated resistance mechanisms. Further translational and clinical research will be essential to refine therapeutic stratification and improve long-term outcomes in epithelial ovarian cancer.

## References

[1] Smolarz B Biernacka K Łukasiewicz H Samulak D Piekarska E Romanowicz H . Ovarian cancer—epidemiology, classification, pathogenesis, treatment, and estrogen receptors’ molecular backgrounds. Int J Mol Sci. (2025) 26:4611. doi: 10.3390/ijms26104611 40429755 PMC12111435

[2] Marconato N De Tommasi O Paladin D Boscarino D Spagnol G Saccardi C . Unraveling homologous recombination deficiency in ovarian cancer: a review of currently available testing platforms. Cancers. (2025) 17:1771. doi: 10.3390/cancers17111771 40507252 PMC12153926

[3] Kausar MA Alshammari KF Alenazi F Anwar S Khalifa AM Ginawi T . *RAD51* and *PALB2* in precision oncology: clinical implications for HRD associated breast and ovarian cancers (review). Int J Oncol. (2025) 67:1–16. doi: 10.3892/ijo.2025.5771 40613200 PMC12270392

[4] Cruz GI Kane KL Matsuno RK Sweetnam C Williams AFO Toland LM . Treatment, outcomes, and resource utilization among patients with metastatic breast and advanced epithelial ovarian cancer, by BRCA1/2 and HRD status. Oncologist. (2025) 30:oyaf100. doi: 10.1093/oncolo/oyaf100 40796115 PMC12342895

[5] Moore K Colombo N Scambia G Kim BG Oaknin A Friedlander M . Maintenance olaparib in patients with newly diagnosed advanced ovarian cancer. N Engl J Med. (2018) 379:2495–505. doi: 10.1056/NEJMoa1810858 30345884

[6] González-Martín A Pothuri B Vergote I DePont Christensen R Graybill W Mirza MR . Niraparib in patients with newly diagnosed advanced ovarian cancer. N Engl J Med. (2019) 381:2391–402. doi: 10.1056/NEJMoa1910962 31562799

[7] Ray-Coquard I Pautier P Pignata S Pérol D González-Martín A Berger R . Olaparib plus bevacizumab as first-line maintenance in ovarian cancer. N Engl J Med. (2019) 381:2416–28. doi: 10.1056/NEJMoa1911361 31851799

[8] Coleman RL Oza AM Lorusso D Aghajanian C Oaknin A Dean A . Rucaparib maintenance treatment for recurrent ovarian carcinoma after response to platinum therapy (ARIEL3): a randomised, double-blind, placebo-controlled, phase 3 trial. Lancet. (2017) 390:1949–61. doi: 10.1016/S0140-6736(17)32440-6 28916367 PMC5901715

[9] Shah B Hussain M Seth A . Homologous recombination deficiency in ovarian and breast cancers: biomarkers, diagnosis, and treatment. Curr Issues Mol Biol. (2025) 47:638. doi: 10.3390/cimb47080638 40864792 PMC12384633

[10] Kulkarni S Seneviratne N Tosun Ç MadhuSudan S . PARP inhibitors in ovarian cancer: mechanisms of resistance and implications to therapy. DNA Repair. (2025) 149:103830. doi: 10.1016/j.dnarep.2025.103830 40203475

[11] Goldlust IS Guidice E Lee J . PARP inhibitors in ovarian cancer. Semin Oncol. (2024) 51:45–57. doi: 10.1053/j.seminoncol.2024.01.001 38262776 PMC11031289

[12] Pepe F Russo G Cefaliello A Lamia MR Buonaiuto R Crimaldi G . Homologous recombination deficiency testing in ovarian cancer: the diagnostic experience of a referral Italian institution. Pathologica. (2025) 117:258–68. doi: 10.32074/1591-951X-1098 40626669 PMC12236138

[13] Zhan Z Zhang J Liang H Wang C Hong L Liu W . KAT6A condensates impair PARP1 trapping of PARP inhibitors in ovarian cancer. Adv Sci. (2024) 11:2400140. doi: 10.1002/advs.202400140 38973255 PMC11425913

[14] Biegała Ł Gajek A Marczak A Rogalska A . PARP inhibitor resistance in ovarian cancer: underlying mechanisms and therapeutic approaches targeting the ATR/CHK1 pathway. Biochim Biophys Acta BBA - Rev Cancer. (2021) 1876:188633. doi: 10.1016/j.bbcan.2021.188633 34619333

[15] Pettitt SJ Krastev DB Brandsma I Dréan A Song F Aleksandrov R . Genome-wide and high-density CRISPR-Cas9 screens identify point mutations in PARP1 causing PARP inhibitor resistance. Nat Commun. (2018) 9:1849. doi: 10.1038/s41467-018-03917-2 29748565 PMC5945626

[16] Ma H Qi G Han F Lu W Peng J Li R . HMGB3 promotes PARP inhibitor resistance through interacting with PARP1 in ovarian cancer. Cell Death Dis. (2022) 13:263. doi: 10.1038/s41419-022-04670-7 35332131 PMC8948190

[17] Venegas L Lheureux S . Interplay of replication stress response and immune microenvironment in high-grade serous ovarian cancer. Front Cell Dev Biol. (2025) 13:1638964. doi: 10.3389/fcell.2025.1638964 40977887 PMC12446242

[18] Pilié PG Tang C Mills GB Yap TA . State-of-the-art strategies for targeting the DNA damage response in cancer. Nat Rev Clin Oncol. (2019) 16:81–104. doi: 10.1038/s41571-018-0114-z 30356138 PMC8327299

[19] Huang TT Chiang CY Nair JR Wilson KM Cheng K Lee JM . AKT1 interacts with DHX9 to mitigate R loop–induced replication stress in ovarian cancer. Cancer Res. (2024) 84:887–904. doi: 10.1158/0008-5472.CAN-23-1908 38241710 PMC10947874

[20] Acharya G Mani C Sah N Saamarthy K Young R Reedy MB . CHK1 inhibitor induced PARylation by targeting PARG causes excessive replication and metabolic stress and overcomes chemoresistance in ovarian cancer. Cell Death Discov. (2024) 10:278. doi: 10.1038/s41420-024-02040-0 38862485 PMC11166985

[21] Qiu Z Singh D Liu Y Prasad CB Bean N Yan C . Low *PPP2R2A* expression promotes sensitivity to CHK1 inhibition in high-grade serous ovarian cancer. Theranostics. (2024) 14:7450–69. doi: 10.7150/thno.96879 39659585 PMC11626944

[22] Liu C Li J Xu F Chen L Ni M Wu J . PARP1-DOT1L transcription axis drives acquired resistance to PARP inhibitor in ovarian cancer. Mol Cancer. (2024) 23:111. doi: 10.1186/s12943-024-02025-8 38778348 PMC11110363

[23] Haynes B Murai J Lee JM . Restored replication fork stabilization, a mechanism of PARP inhibitor resistance, can be overcome by cell cycle checkpoint inhibition. Cancer Treat Rev. (2018) 71:1–7. doi: 10.1016/j.ctrv.2018.09.003 30269007 PMC7429716

[24] Lin KK Harrell MI Oza AM Oaknin A Ray-Coquard I Tinker AV . *BRCA* reversion mutations in circulating tumor DNA predict primary and acquired resistance to the PARP inhibitor rucaparib in high-grade ovarian carcinoma. Cancer Discov. (2019) 9:210–9. doi: 10.1158/2159-8290.CD-18-0715 30425037

[25] Collet L Hanvic B Turinetto M Treilleux I Chopin N Le Saux O . BRCA1/2 alterations and reversion mutations in the area of PARP inhibitors in high grade ovarian cancer: state of the art and forthcoming challenges. Front Oncol. (2024) 14:1354427. doi: 10.3389/fonc.2024.1354427 38544832 PMC10965616

[26] Guffanti F Mengoli I Damia G . Current HRD assays in ovarian cancer: differences, pitfalls, limitations, and novel approaches. Front Oncol. (2024) 14:1405361. doi: 10.3389/fonc.2024.1405361 39220639 PMC11361952

[27] Tao L Zhou Y Pan X Luo Y Qiu J Zhou X . Repression of LSD1 potentiates homologous recombination-proficient ovarian cancer to PARP inhibitors through down-regulation of BRCA1/2 and RAD51. Nat Commun. (2023) 14:7430. doi: 10.1038/s41467-023-42850-x 37973845 PMC10654398

[28] Sun C Li X Teng Q Liu X Song L Schiöth HB . Targeting platinum-resistant ovarian cancer by disrupting histone and RAD51 lactylation. Theranostics. (2025) 15:3055–75. doi: 10.7150/thno.104858 40083924 PMC11898288

[29] Song H Dicks E Ramus SJ Tyrer JP Intermaggio MP Hayward J . Contribution of germline mutations in the *RAD51B*, *RAD51C*, and *RAD51D* genes to ovarian cancer in the population. J Clin Oncol. (2015) 33:2901–7. doi: 10.1200/JCO.2015.61.2408 26261251 PMC4554751

[30] Li H Liu ZY Wu N Chen YC Cheng Q Wang J . PARP inhibitor resistance: the underlying mechanisms and clinical implications. Mol Cancer. (2020) 19:107. doi: 10.1186/s12943-020-01227-0 32563252 PMC7305609

[31] Littler S Barnes BM Owen R Nelson L Tighe A Lin IH . Targeting SUMOylation in ovarian cancer: sensitivity, resistance, and the role of MYC. iScience. (2025) 28:112555. doi: 10.1016/j.isci.2025.112555 40487451 PMC12145850

[32] Vaidyanathan A Sawers L Gannon AL Chakravarty P Scott AL Bray SE . ABCB1 (MDR1) induction defines a common resistance mechanism in paclitaxel- and olaparib-resistant ovarian cancer cells. Br J Cancer. (2016) 115:431–41. doi: 10.1038/bjc.2016.203 27415012 PMC4985349

[33] Wang F Guo Z Carr MJ Shi W . PARPs and PARP inhibitors: molecular mechanisms and clinical applications. Mol BioMed. (2025) 6:152. doi: 10.1186/s43556-025-00385-1 41460301 PMC12748464

[34] Ma X Qu L Wu Q Yu Q Lin Z Yang Y . Advances in ovarian cancer: biological insights, therapeutic innovations, and future perspectives. J Ovarian Res. (2025) 19:22. doi: 10.1186/s13048-025-01921-x 41354809 PMC12829294

[35] Kyo S Kanno K Takakura M Yamashita H Ishikawa M Ishibashi T . Clinical landscape of PARP inhibitors in ovarian cancer: molecular mechanisms and clues to overcome resistance. Cancers. (2022) 14:2504. doi: 10.3390/cancers14102504 35626108 PMC9139943

[36] Sun Y Rodgers Furones A Gultekin O Khare S Neo SY Shi W . Adaptive NK cells exhibit tumor-specific immune memory and cytotoxicity in ovarian cancer. Cancer Immunol Res. (2025) 13:1080–97. doi: 10.1158/2326-6066.CIR-24-0852 40293356

[37] Xiao H Cheng G Zhang H Liu Y Chen Z Gao Y . Role of KLF5 in enhancing ovarian cancer stemness and PARPi resistance: mechanisms and therapeutic targeting. J Transl Med. (2025) 23:492. doi: 10.1186/s12967-025-06502-6 40307891 PMC12042437

[38] Cordani N Bianchi T Ammoni LC Cortinovis DL Cazzaniga ME Lissoni AA . An overview of PARP resistance in ovarian cancer from a molecular and clinical perspective. Int J Mol Sci. (2023) 24:11890. doi: 10.3390/ijms241511890 37569269 PMC10418869

[39] Wu H Sun C Cao W Teng Q Ma X Schiöth HB . Blockade of the lncRNA-PART1-PHB2 axis confers resistance to PARP inhibitor and promotes cellular senescence in ovarian cancer. Cancer Lett. (2024) 602:217192. doi: 10.1016/j.canlet.2024.217192 39181433

[40] Ding L Wang Q Martincuks A Kearns MJ Jiang T Lin Z . STING agonism overcomes STAT3-mediated immunosuppression and adaptive resistance to PARP inhibition in ovarian cancer. J Immunother Cancer. (2023) 11:e005627. doi: 10.1136/jitc-2022-005627 36609487 PMC9827255

[41] Liu D Tao K Xu C Yang W Cai C Feng C . Tumor cell–derived IFN spatially reprograms osteopontin-enriched macrophage niches to promote PARP inhibitor resistance. J Clin Invest. (2026) 136:e199035. doi: 10.1172/JCI199035 41734034 PMC13078868

[42] Tarannum M Dinh K Vergara J Birch G Abdulhamid YZ Kaplan IE . CAR memory-like NK cells targeting the membrane proximal domain of mesothelin demonstrate promising activity in ovarian cancer. Sci Adv. (2024) 10:eadn0881. doi: 10.1126/sciadv.adn0881 PMC11244547 38996027

[43] Kim H Xu H George E Hallberg D Kumar S Jagannathan V . Combining PARP with ATR inhibition overcomes PARP inhibitor and platinum resistance in ovarian cancer models. Nat Commun. (2020) 11:3726. doi: 10.1038/s41467-020-17127-2 32709856 PMC7381609

[44] Zou Y Zhang H Chen P Tang J Yang S Nicot C . Clinical approaches to overcome PARP inhibitor resistance. Mol Cancer. (2025) 24:156. doi: 10.1186/s12943-025-02355-1 40442774 PMC12123805

[45] Garg V Oza AM . Treatment of ovarian cancer beyond PARP inhibition: current and future options. Drugs. (2023) 83:1365–85. doi: 10.1007/s40265-023-01934-0 37737434 PMC10581945

[46] Bi R Chen L Huang M Qiao Z Li Z Fan G . Emerging strategies to overcome PARP inhibitors’ resistance in ovarian cancer. Biochim Biophys Acta BBA - Rev Cancer. (2024) 1879:189221. doi: 10.1016/j.bbcan.2024.189221 39571765

[47] Soberanis Pina P Lheureux S . Overcoming PARP inhibitor resistance in ovarian cancer. Int J Gynecol Cancer. (2023) 33:364–76. doi: 10.1136/ijgc-2022-003698 36878566

[48] McMullen M Karakasis K Madariaga A Oza AM . Overcoming platinum and PARP-inhibitor resistance in ovarian cancer. Cancers. (2020) 12:1607. doi: 10.3390/cancers12061607 32560564 PMC7352566

[49] Lheureux S Prokopec SD Oldfield LE Gonzalez-Ochoa E Bruce JP Wong D . Identifying mechanisms of resistance by circulating tumor DNA in EVOLVE, a phase II trial of cediranib plus olaparib for ovarian cancer at time of PARP inhibitor progression. Clin Cancer Res. (2023) 29:3706–16. doi: 10.1158/1078-0432.CCR-23-0797 37327320 PMC10502468

[50] Han F Qi G Li R Peng J Yan S Yuan C . USP28 promotes PARP inhibitor resistance by enhancing SOX9-mediated DNA damage repair in ovarian cancer. Cell Death Dis. (2025) 16:305. doi: 10.1038/s41419-025-07647-4 40240356 PMC12003857

[51] Pujade-Lauraine E Selle F Scambia G Asselain B Marmé F Lindemann K . Maintenance olaparib rechallenge in patients with platinum-sensitive relapsed ovarian cancer previously treated with a PARP inhibitor (OReO/ENGOT-ov38): a phase IIIb trial. Ann Oncol. (2023) 34:1152–64. doi: 10.1016/j.annonc.2023.09.3110 37797734

[52] Shah PD Wethington SL Pagan C Latif N Tanyi J Martin LP . Combination ATR and PARP inhibitor (CAPRI): a phase 2 study of ceralasertib plus olaparib in patients with recurrent, platinum-resistant epithelial ovarian cancer. Gynecol Oncol. (2021) 163:246–53. doi: 10.1016/j.ygyno.2021.08.024 34620496 PMC9614917

[53] Banerjee S Stewart J Porta N Toms C Leary A Lheureux S . ATARI trial: ATR inhibitor in combination with olaparib in gynecological cancers with ARID1A loss or no loss (ENGOT/GYN1/NCRI). Int J Gynecol Cancer. (2021) 31:1471–5. doi: 10.1136/ijgc-2021-002973 34518240 PMC8573414

[54] Westin SN Coleman RL Fellman BM Yuan Y Sood AK Soliman PT . EFFORT: EFFicacy Of adavosertib in parp ResisTance: a randomized two-arm non-comparative phase II study of adavosertib with or without olaparib in women with PARP-resistant ovarian cancer. J Clin Oncol. (2021) 39:5505. doi: 10.1200/JCO.2021.39.15_suppl.550542586871

[55] Drew Y Kim JW Penson RT O'Malley DM Parkinson C Roxburgh P . Olaparib plus Durvalumab, with or without Bevacizumab, as treatment in PARP inhibitor-naïve platinum-sensitive relapsed ovarian cancer: a phase II multi-cohort study. Clin Cancer Res. (2024) 30:50–62. doi: 10.1158/1078-0432.CCR-23-2249 37939124 PMC10767301

[56] Harter P Trillsch F Okamoto A Reuss A Kim JW Rubio-Pérez MJ . Durvalumab with carboplatin/paclitaxel and bevacizumab followed by durvalumab and bevacizumab with or without olaparib maintenance in newly diagnosed non-BRCA-mutated advanced ovarian cancer. Ann Oncol. (2026) 37:503–20. doi: 10.1016/j.annonc.2025.11.020 41380962

[57] Vergote I Sehouli J Salutari V Zola P Madry R Wenham RM . ENGOT-OV43/KEYLYNK-001: a phase III, randomized, double-blind, active- and placebo-controlled study of pembrolizumab plus chemotherapy with olaparib maintenance for first-line treatment of BRCA-nonmutated advanced epithelial ovarian cancer. J Clin Oncol. (2019) 37:TPS5603–TPS5603. doi: 10.1200/JCO.2019.37.15_suppl.TPS560342586871

[58] Lee JM Moore RG Ghamande S Park MS Diaz JP Chapman J . Cediranib in combination with olaparib in patients without a germline BRCA1/2 mutation and with recurrent platinum-resistant ovarian cancer: phase IIb CONCERTO trial. Clin Cancer Res. (2022) 28:4186–93. doi: 10.1158/1078-0432.CCR-21-1733 35917514 PMC9527502

[59] De Tommasi O Pan H Spagnol G Massa D Marconato N Incognito GG . New frontiers in the management of recurrent epithelial ovarian cancer: a comprehensive review. Gynecol Obstet Clin Med. (2025) 5:e000277. doi: 10.1136/gocm-2025-000277

